# Effects of heterozygosity on performance of purebred and crossbred pigs

**DOI:** 10.1186/s12711-019-0450-1

**Published:** 2019-02-28

**Authors:** Maja Winther Iversen, Øyvind Nordbø, Eli Gjerlaug-Enger, Eli Grindflek, Marcos Soares Lopes, Theo Meuwissen

**Affiliations:** 1 Norsvin R&D, Storhamargata 44, 2317 Hamar, Norway; 2 Norwegian University of Life Sciences, Postboks 5003 NMBU, 1432 Ås, Norway; 3 GENO SA, Storhamargata 44, 2317 Hamar, Norway; 4 Topigs Norsvin Research Center, 6641 SZ Beuningen, The Netherlands; 5 Topigs Norsvin, Curitiba, 80420-210 Brazil

## Abstract

**Background:**

In pigs, crossbreeding aims at exploiting heterosis, but heterosis is difficult to quantify. Heterozygosity at genetic markers is easier to measure and could potentially be used as an indicator of heterosis. The objective of this study was to investigate the effect of heterozygosity on various maternal and production traits in purebred and crossbred pigs. The proportion of heterozygosity at genetic markers across the genome for each individual was included in the prediction model as a fixed regression across or within breeds.

**Results:**

Estimates of regression coefficients of heterozygosity showed large effects for some traits. For maternal traits, regression coefficient estimates were always in a favourable direction, while for production, meat and slaughter quality traits, they were both favourable and unfavourable. Traits with the largest estimated effects of heterozygosity were total number born, litter weight at 3 weeks, weight at 150 days, and age at 40 kg. Estimates of regression coefficients on heterozygosity differed between breeds. Traits with the largest effect of heterozygosity also showed a significant (P < 0.05) increase in prediction accuracy when heterozygosity was included in the model compared to the model without heterozygosity.

**Conclusions:**

For traits with the largest estimates of regression coefficients on heterozygosity, the inclusion of heterozygosity in the model improved prediction accuracy. Using models that include heterozygosity would result in selecting different animals for breeding, which has the potential to improve genetic gain for these traits. This is most beneficial when crossbreds or several breeds are included in the estimation of breeding values and is relevant to all species, not only pigs. Thus, our results show that including heterozygosity in the model is beneficial for some traits, likely due to dominant gene action.

**Electronic supplementary material:**

The online version of this article (10.1186/s12711-019-0450-1) contains supplementary material, which is available to authorized users.

## Background

One of the main reasons for producing crossbreds (CB) in animal breeding programs is to take advantage of heterosis (“the difference between crossbred and inbred means” [1]), but heterosis can be difficult to estimate. However, heterozygosity at genetic markers is much easier to measure (in genotyped individuals). Although heterozygosity is not the same as heterosis, heterozygosity may be a useful indicator when predicting the ability of purebreds (PB) to produce good CB offspring. Zhang et al. [2] found a significant correlation between heterosis and individual heterozygosity for birth weight in an F1 cross of pigs, but not for average daily gain and feed conversion ratio in the same population. In addition, when dominant gene action, which results in differences between the genotypic value and the breeding value at a single locus [1], is present, including heterozygosity in the model as an alternative to fitting a dominance model [3] would be interesting because it is less computationally demanding. An alternative approach is to include inbreeding in the model, since it has the opposite effect of heterozygosity on phenotype(s) (e.g. if increased heterozygosity increases the phenotype, inbreeding would decrease it), and Xiang et al. [4] found that the predictive ability for total number born was improved when including (genomic) inbreeding in the model. The level of heterozygosity of an individual is very easy to obtain from genotypes and requires no knowledge of ancestry, in contrast to pedigree inbreeding. In general, the level of heterozygosity is expected to be higher in CB than in PB animals and individuals with higher heterozygosity are expected to have better fitness. An individual’s average heterozygosity across markers can be used as a measure of heterozygosity. An alternative would be to use standardized heterozygosity, for which a common divisor is used for each defined group of individuals [5]. In this case, animals from different breeds or that were genotyped on different single nucleotide polymorphism (SNP) chips could be compared by standardizing the heterozygosity, i.e. dividing the heterozygosity by the mean within the group. Against this background, the objective of our study was to investigate the effect of heterozygosity on various traits in PB and CB animals and determine whether inclusion of heterozygosity in models for the estimation of breeding values can increase prediction accuracy, both in CB and their PB parents.

## Methods

### Animals and data

Three sets of data were available for this study (Table 1): two were provided by Norsvin SA (Hamar, Norway, datasets 1 and 3) and one by Topigs Norsvin (Beuningen, the Netherlands, dataset 2). Dataset 1 included data from Norwegian Landrace, dataset 2 from Dutch Landrace (breed A), Dutch Large White (breed B), and their F1 cross (breed X), and dataset 3 from a synthetic sire line that was composed of two synthetic sire lines (Lines 1 and 2) that have different genetic origins and have been under selection independently from each other. Dataset 3 contains animals of the two original lines, their F1 cross, and a backcross (Line 1 × F1). Datasets 1 and 2 comprised maternal traits and dataset 3 comprised production, meat quality, and slaughter quality traits (and one maternal trait) (Table 1). Not all animals had observations on all traits and only females had observations on maternal traits.

**Table 1:** Description of traits and numbers of observations for the three datasets

Dataset	Breed	Animals with genotypes^b^	Animals with both genotypes and phenotypes
1	Norwegian Landrace	22,558	13,283
2	Dutch Landrace (A)	3238	2377
2	Dutch Large White (B)	3735	2859
2	F1-cross^a^ (X)	1377	1312
3	Synthetic sire line^c^	19,477	19,135
*Dataset 1*—*Norwegian Landrace*			
Trait	Trait description	Observations^d^	Animals
TNB	Total number born	28,790	13,279
SB	Number of stillborn piglets	28,790	13,279
D3	Number of dead piglets at 3 weeks	15,573	9476
LW3	Litter weight at 3 weeks in kg	16,301	9734
VAR3W	Variance in weight within litter at 3 weeks	16,138	9619
SL	Shoulder lesions of sow at weaning	23,466	12,521
BCS	Body condition score of sow at weaning	23,463	12,521
*Dataset 2*—*Dutch Landrace, Large White, and F1*			
Trait	Trait description	Observations^d^	Animals
TNB	Total number born	35,852	6507
LB	Piglets born alive	35,812	6507
GL	Gestation length in days	30,559	5844
*Dataset 3*—*Synthetic sire line*			
Trait	Trait description	Observations	Animals
W21	21-day weight in kg	11,994	11,994
W150	150-day weight in kg	11,029	11,029
BF100	Backfat at 100 kg measured on live animals in mm	14,407	14,407
LD100	Loin depth at 100 kg measured on live animals in mm	13,844	13,844
A40	Age at 40 kg in days	16,480	16,480
DTP	Days test period at boar station (days from 40-120 kg)	16,314	16,314
TFI	Total feed intake from 40-120 kg in kg (i.e. feed intake per 80 kg live weight gain)	11,947	11,947
LMP	Lean meat percentage	7476	7476
DP	Dressing percentage (slaughter weight/live weight)	7475	7475
IMF	Intramuscular fat percentage measured in the laboratory (g/100 g)	3379	3379
PHL	pH of loin	3640	3640
DRIP	Drip loss is the percentage loss of water from a piece of loin muscle between 96 h post mortem to 120 h post mortem	3615	3615
LB1	Live born first parity	4921	4921

^a^F1 cross of Dutch Landrace and Large White

^b^Number of animals included in the genomic relationship matrix

^c^The synthetic line is a sire line made of two lines of the same breed (Line 1 and 2) that have been under selection independently from each other. The dataset contains both animals of the two original lines, their F1 and a backcross (Line 1 × F1)

^d^Maternal traits have repeated observations for most animals

Genotypes for the breeds were obtained from different SNP chips. Genotyping was performed at CIGENE (University of Life Sciences, Ås, Norway) or at GeneSeek (Lincoln, NE, USA), using either the Illumina GeneSeek custom 80 K SNP chip (Lincoln, NE, USA), the Illumina Porcine SNP60 Beadchip (Illumina Inc., San Diego, CA, USA), an Illumina GeneSeek custom 50 K SNP chip (Lincoln, NE, USA), an Illumina GeneSeek custom 10 K SNP chip (Lincoln, NE, USA), or the Illumina Porcine SNP9 Beadchip (Illumina Inc., San Diego, CA, USA). Genome positions of the SNPs were based on the Sscrofa10.2 assembly of the reference genome [6].

Genotyping of Norwegian Landrace animals (dataset 1) was performed on the 80 K (N = 11,239), 60 K (N = 8188), 50 K (N = 2130), and 9 K (N = 1043) SNP chips (as defined above). Before imputation, the SNPs were filtered within chip by excluding SNPs with a call rate lower than 0.85, a minor allele frequency (MAF) lower than 0.001, and strong deviations from Hardy–Weinberg equilibrium (P < 1 × 10^−7^). The low MAF threshold of 0.001 was used to allow for investigation of SNPs with a low MAF, but a MAF threshold of 0.01 was used to build the genomic relationship matrix. Genotypes of animals that had missing genotypes for more than 30% of SNPs were excluded and, to avoid outliers due to admixing of biological samples, genotypes of animals with a heterozygosity that deviated more than 3 times the interquartile distance from the mean heterozygosity of the breed were also excluded. Only SNPs located on the 18 autosomal chromosomes and that were present on the Illumina Porcine SNP50 Beadchip were included in the imputation. Imputation was performed using AlphaImpute [7], which combines a heuristic approach and a hidden Markov model [8] and which imputes all missing genotypes. After imputation, genotypes on 37,206 SNPs were available.

For the Dutch Landrace, Dutch Large White animals and their F1 cross (dataset 2), both male and female PB were genotyped, but in the F1 population, only females were genotyped (same procedure as in [9]). All these animals were genotyped using the 60 K SNP chip (as defined above). Quality control was done separately for each breed and consisted of excluding SNPs with a GenCall lower than 0.15, a call rate lower than 0.95, a MAF lower than 0.01, and strong deviations from Hardy–Weinberg equilibrium (P < 1 × 10^−12^). SNPs located on sex chromosomes and unmapped SNPs were also excluded. All genotyped animals had a frequency of missing genotypes lower than 0.05 and were therefore kept for further analyses. After quality control, the remaining missing genotypes were imputed using Fimpute V2.2 [10] and SNPs that did not segregate in all breeds for dataset 2 were excluded, leaving 36,778 SNPs that were common to all breeds for further analysis.

For the synthetic sire line (dataset 3), genotyping was performed using the 80 K (N = 8134), 60 K (N = 6360), 50 K (N = 3245) or 10 K (N = 1738) SNP chip (as defined above). Quality control consisted of excluding SNPs with a GenCall lower than 0.15 (Illumina Inc., 2005), a call rate lower than 0.95, a MAF lower than 0.02, strong deviations from Hardy–Weinberg equilibrium (P < 1 × 10^−12^), SNPs located on sex chromosomes, and unmapped SNPs. All genotyped animals had a frequency of missing genotypes lower than 0.05 and were therefore kept for further analyses. After quality control, the remaining missing genotypes of the animals genotyped with the 80 K SNP chip were imputed using Fimpute V2.2 [10] and animals genotyped with the 60 K, 50 K and 10 K SNP chips had their genotypes imputed to the 80 K SNP chip. The final dataset consisted of genotypes on 41,208 SNPs from the 80 K SNP chip.

The reason for using different parameters, SNP densities, and imputation software was due to practical and historical reasons. The SNP data were taken directly from routine genomic evaluations for different breeds in Topigs Norsvin, and different quality checks and imputation pipelines were developed before the merge of Topigs and Norsvin International in 2014. Since the SNP data was part of industrial routine genomic evaluations and different research projects, parameters were well tuned and optimized within each breed to maximize genetic gain. We are confident that the different SNP densities and the use of two different software (Alphaimpute and Fimpute) for imputation did not compromise our results because it has been shown that a small number of SNPs (N = 2000) can reproduce results obtained from higher SNP densities (60 K) [11] in a GBLUP context and that both software result in similar imputation accuracies [12].

### Statistical analysis

Heterozygosity was measured as the proportion of heterozygous marker genotypes for each individual. Thus, only genotyped individuals had a heterozygosity observation. Heterozygosity was also modelled within breed, when appropriate. Expected heterosis was used for the synthetic sire line and was calculated from the available pedigree. PB animals (Lines 1 and 2) were given an expected heterosis of 0%, F1 animals have an expected heterosis of 100%, and backcrosses have an expected heterosis of 50%, etc.

Four models were compared across datasets. Model 1 (base model) was the model used in routine evaluations in Norsvin and Topigs Norsvin for each trait (see Additional file 1: Tables S1, S2, and S3). The models used in routine evaluations do not consider heterosis or inbreeding, and are breed-specific, but for dataset 3 considers line composition. For Models 2, 3 and 4, heterozygosity, heterozygosity within breed, or expected heterosis was added as a fixed regression in addition to the original base model (Table 2). Table 2 gives an overview of which models were used for each dataset. Fixed regressions cannot have missing values, so only genotyped animals were included, and consequently the models were used with GBLUP in the MiXBLUP software [13].

**Table 2:** Models used for each dataset

Model^a^	Dataset 1	Dataset 2	Dataset 3
1 Base model	X	X	X
2 Base model + heterozygosity	X	X	X
3 Base model + heterozygosity(breed)^b^		X	X
4 Base model + expected heterosis^c^			X

Dataset 1 included data from Norwegian Landrace, dataset 2 from Dutch Landrace (breed A), Dutch Large White (breed B), and their F1 cross (breed X), and dataset 3 from a synthetic sire line that was composed of two synthetic sire lines (Lines 1 and 2) that have different genetic origins and have been under selection independently from each other

^a^‘Base’ is the base model for each trait from routine evaluations, without any heterozygosity effects, and the remaining models have one heterozygosity effect as indicated

^b^For the synthetic line, breed is proportion of Line 1 (one of the original lines for the synthetic line). This information was available from the pedigree

^c^Purebred animals have an expected heterosis of 0%, F1 have an expected heterosis of 100%, backcrosses (Line 1 x F1) have an expected heterosis of 50%, etc. Values were based on existing pedigree data for the synthetic sire line

Regression coefficients on heterozygosity (and other heterozygosity factors) were extracted from the *Solreg* and *Solfix* files from MiXBLUP using all data within each dataset for each model. Standard errors for regression coefficients are not available from MiXBLUP. Therefore, models were re-run in DMU4 [14]. Due to limited capacity in DMU, dataset 1 was analysed only with single-trait models and dataset 3 was analysed only with pedigree, with five, four and four traits included in multiple trait models (grouped in the same order as in Table 1). Thus, the standard errors obtained are not exact for the regression coefficients from MiXBLUP and results should be interpreted with caution. For dataset 2, it was still possible to run a multiple trait model with genomic information in DMU. To estimate the effect of heterozygosity on each trait in comparison to other traits, the regression coefficients for heterozygosity were divided by the phenotypic standard deviation for the traits. Models were also compared based on rank correlations of animals according to their estimated breeding value (EBV) for each model. To determine whether different animals were selected with different models, the correlation between the rank of the top 100 (validation) animals for one model with the rank of the same 100 animals for a second model were estimated in pairwise comparisons between models, i.e. the top 100 animals from Model 1 are not necessarily the same as the top 100 animals from Model 3. Thus, the correlation was estimated twice for each pairwise model comparison.

Comparisons of models were performed with correlations between yield deviations (YD) and EBV. Predictive ability was calculated as: ${\text{cor}}\left[ {\left( {{\text{YD }} + {\text{hetReg}}} \right), \left( {{\text{GEBV }} + {\text{hetReg}}} \right)} \right]$, where ${\text{YD}}$ is the (mean) yield deviation of the animal as estimated by MiXBLUP [13] by each model, ${\text{hetReg}}$ is the heterozygosity factor multiplied by its estimated regression coefficient, and ${\text{GEBV}}$ is the genomic EBV. The ${\text{hetReg}}$ part of the equation was included to make models comparable to the base model because, for Models 2, 3 and 4 (Table 2), it is removed from both ${\text{YD}}$ and EBV, i.e. fixed, non-genetic random, and regression effects (including heterozygosity effects) are subtracted from the phenotype to estimate ${\text{YD}}$ for these models, as shown in the following:

$$\begin{aligned}{\mathbf{y}} = {\mathbf{Xb}} + {\mathbf{hetReg}} + {\mathbf{Nn}} + {\mathbf{GEBV}} + {\mathbf{e}},\end{aligned}$$

$$\begin{aligned}{\mathbf{y}} - {\mathbf{Xb}} - {\mathbf{hetReg}} - {\mathbf{Nn}} = {\mathbf{GEBV}} + {\mathbf{e}},\end{aligned}$$

$$\begin{aligned}{\mathbf{YD}} = {\mathbf{GEBV}} + {\mathbf{e}}, \end{aligned}$$

where ${\mathbf{y}}$ is the phenotype for an animal, ${\mathbf{Xb}}$ are fixed effects and covariates, ${\text{hetReg}}$ is as defined above, ${\mathbf{Nn}}$ is the non-genetic random effect, ${\text{GEBV}}$ is the genomic estimated breeding value, and ${\mathbf{e}}$ is the residual. The ${\text{hetReg}}$ part of the equation was not included in the base model because it did not include heterozygosity effects.

The 5000 youngest animals with both genotypes and phenotypes were used for validation for datasets 1 and 3. Because dataset 2 was too small, instead, two validation sets were created for each of the PB, and one for the CB, with 1000 animals each. For the first validation set, the 1000 youngest animals were removed from the training set. For the second validation set, these were added back to the training set, and the next 1000 animals were removed. This was done separately for each breed (the other breeds were still in the dataset). For the crossbreds, only one validation set was used (1000 youngest animals) due to the limited number of available animals.

To test whether the models differed significantly in predictive ability, we used a bootstrap procedure [15] for the correlation between ${\text{YD}}$ (+ ${\text{hetReg}}$) and ${\text{GEBV}}$ (+ ${\text{hetReg}}$) for each model. A pairwise comparison of ${\text{GEBV}}$ from two models at a time was performed to determine which of the two models predicted its ${\text{YD}}$ best by randomly sampling data point quadruplets with replacement: the ${\text{YD}}$ and their predictions (${\text{GEBV}}$) using two models. A total of 10,000 bootstrap samples were constructed for each pairwise comparison. The size of the input data for the bootstrap procedure was the same as for the validation sets. For each bootstrap sample, we determined for which model the ${\text{GEBV}}$ yielded a greater correlation with the ${\text{YD}}$ of that model. By counting the number of times one model had a higher correlation than the other, the two models were considered as significantly different if one of the models had a higher correlation in at least 97.5% of the bootstrap samples (tests at a P value of 5% due to the two-sided nature of the test). This is similar to bootstrapping methods used by others [16, 17].

## Results

Please note that part of the results from this study were presented at the 2018 World Congress of Genetics Applied to Livestock Production in Auckland, New Zealand [18]. These were the estimated regression coefficients for datasets 1 and 2 and prediction accuracies without indications on significant differences. There was, however, an error in prediction accuracies, which has been corrected in this paper.

Descriptive statistics for the traits for the three datasets are in Table 3. Separate means per breed/line for datasets 2 and 3 are in Tables S4 and S5 (see Additional file 2: Tables S4 and S5).

**Table 3:** Descriptive statistics for the three datasets

Dataset^a^	Trait^b^	Mean	SD	Minimum	Maximum	h^2^
1	TNB	14.00	3.42	1.00	26.00	0.08
1	SB	1.22	1.54	0.00	18.00	0.07
1	D3	1.68	1.78	0.00	20.00	0.06
1	LW3, kg	76.78	18.74	2.50	152.40	0.13
1	VAR3W	1.10	0.39	0.07	3.77	0.06
1	SL	0.41	0.82	0.00	4.00	0.13
1	BCS	4.15	0.94	1.00	9.00	0.13
2	TNB	15.34	3.46	1.00	32.00	0.10
2	LB	14.02	3.23	1.00	28.00	0.07
2	GL, d	115.48	1.65	105.00	124.00	0.34
3	W21, kg	6.92	1.43	2.26	12.59	0.06
3	W150, kg	108.98	11.70	52.16	148.74	0.36
3	BF100, mm	7.09	2.19	2.80	16.00	0.43
3	LD100, mm	51.11	9.46	28.30	77.25	0.37
3	A40, d	86.76	7.10	60.01	121.44	0.41
3	DTP, d	77.40	9.69	44.55	136.52	0.42
3	TFI, kg	173.00	16.25	89.60	261.10	0.38
3	LMP, %	61.41	3.46	46.50	74.20	0.59
3	DP, %	71.23	2.57	62.53	81.64	0.27
3	IMF, g/100 g	1.78	0.41	0.00	4.20	0.68
3	PHL	561.60	13.62	513.00	631.00	0.37
3	DRIP, %	3.81	1.79	0.03	11.40	0.32
3	LB1	8.33	2.86	0.00	18.00	0.11

^a^Dataset 1 is Norwegian Landrace, dataset 2 is Dutch Landrace, Dutch Large White and their F1 cross, dataset 3 is the synthetic line

^b^TNB = total number born, SB = stillborn, D3 = number of dead piglets at 3 weeks, LW3 = litter weight at 3 weeks, VAR3W = variance in weight within the litter at 3 weeks, SL = shoulder lesions of sow at weaning, BCS = body condition score of sow at weaning, LB = live born, GL = gestation length, W21 = 21 days weight in kg, W150 = 150 days weight in kg, BF100 = backfat at 100 kg measured on live animals in mm, LD100 = loin depth at 100 kg measured on live animals in mm, A40 = age at 40 kg in days, DTP = days from 40 to 120 kg, TFI = total feed intake from 40 to 120 kg in kg (i.e. feed intake per 80 kg live weight gain), LMP = lean meat percentage, DP = dressing percentage (slaughter weight/live weight), IMF = intramuscular fat percentage measured in the laboratory, PHL = pH of loin, DRIP = drip loss is the percentage loss of water from a piece of loin muscle between 96 h post mortem to 120 h post mortem, LB1 = live born first parity

As expected, CB animals had a greater mean heterozygosity than the PB animals (Table 4), but there were also differences between PB populations, with the Norwegian Landrace having the lowest heterozygosity. This was also expected since this population has been closed for a longer time than the other breeds. In addition, ascertainment biases when choosing the SNP assays for the variety of SNP chips used may have affected the observed heterozygosity for some of the breeds. However, variation in heterozygosity within populations was small, and similar between breeds. Across all breeds, heterozygosity at an individual level ranged from 0.18 to 0.46.

**Table 4:** Mean and standard deviation (SD) of heterozygosity for each population

Dataset	Population	Mean^a^	SD
1	Norwegian Landrace	0.322	0.016
2	Dutch Landrace	0.358	0.024
2	Dutch Large White	0.362	0.017
2	F1-cross^b^	0.426	0.011
3	Synthetic breed^c^	0.355	0.024

^a^Mean of the proportion of heterozygous markers (per individual) in the population

^b^F1 cross of Dutch Landrace and Dutch Large White

^c^The synthetic line is a sire line made of two synthetic sire lines of different genetic origins that have been under selection independently from each other. The dataset contains both animals of the two original lines, their F1 and a backcross (Line 1 × F1)

### Regression coefficients on heterozygosity

For Norwegian Landrace, estimates of the regression coefficient on heterozygosity were in a favourable direction for all traits (i.e. the direction that the traits are selected for in the breeding program, Table 5), i.e. an increase in heterozygosity is expected to change each trait in a favourable direction relative to the breeding goal and production economy. Some regression coefficient estimates were also substantial. For example, a 2.5% increase in heterozygosity was estimated to result in 0.4 more piglets born and a 1.07 kg heavier litter at 3 weeks. When dividing the regression coefficient estimates by the phenotypic standard deviation for the trait, it is possible to get an indication of the importance of heterozygosity across traits. For Norwegian Landrace (Table 5), this indicated that heterozygosity had the largest effect for total number born, litter weight at 3 weeks, and body condition score of the sow at weaning. However, regression coefficient estimates were significantly different from 0 (P < 0.05) only for total number born and litter weight at 3 weeks.

**Table 5:** Estimates of regression coefficients on heterozygosity for Norwegian Landrace for Model 2

Trait	Estimate (SE)	Estimate/σ_p_^a^
Total number born	16.05 (1.60)*	4.69
Stillborn	− 1.73 (0.76)	− 1.12
Dead piglets at 3 weeks	− 0.83 (1.13)	− 0.46
Litter weight at 3 weeks, kg	43.44 (6.10)*	2.32
Litter variance at 3 weeks	− 0.16 (0.24)	− 0.41
Shoulder lesions of sow at weaning	− 0.51 (0.37)	− 0.62
Body condition score of sow at weaning	1.43 (0.44)	1.52

* Regression coefficient significantly different from zero at P < 0.05

^a^Regression coefficient divided by phenotypic standard deviation

For total number born, estimates of regression coefficients (Table 6) on heterozygosity were smaller for Dutch Landrace, Large White, and their F1 cross (dataset 2) than for Norwegian Landrace but they were always in a favourable direction. Estimates of regression coefficients on heterozygosity within breed (Hetbreed) differed a lot between breeds, and the estimates for crossbreds were closer to those for breed B than breed A for total number born and live born, but closer to those for breed A than breed B for gestation length. When comparing estimates of regression coefficients divided by phenotypic standard deviations for dataset 2, the effect of heterozygosity was largest for number of live born and gestation length (Table 6) but the trait with the largest estimated effect of heterozygosity differed between breeds. For both breed B and the CB, the effect of heterozygosity was largest for live born, but for breed A the largest effect was for gestation length. All regression coefficient estimates were significantly different from zero (P < 0.05) for Model 2, but for Model 3, only the estimate for gestation length was significant for breed A and only the estimates for total number of born and live born were significant for breed B. None of the regression coefficient estimates for the F1 were significantly different from zero. The across-breed regression coefficient estimates from Model 2 varied more than the within-breed coefficient estimates from Model 3, which probably resulted in greater power to find statistically significant results for Model 2.

**Table 6:** Estimates of regression coefficients (SE) on heterozygosity for dataset 2 for Models 2 and 3

Trait	Heterozygosity	Hetbreed^b^ (A)^a^	Hetbreed (B)	Hetbreed (X)
*Regression coefficients*				
Total number born	4.22 (1.44)*	1.76 (2.00)	7.14 (2.24)*	6.04 (5.05)
Live born	4.27 (1.34)*	1.89 (1.87)	7.06 (2.09)*	6.16 (4.70)
Gestation length	− 2.07 (0.70)*	− 2.82 (0.97)*	− 1.18 (1.05)	− 2.26 (3.28)
*Standardised regression coefficients* ^c^				
Total number born	1.18	0.52	1.97	1.68
Live born	1.28	0.58	2.11	1.79
Gestation length	− 1.25	− 1.74	− 0.75	− 1.45

* Regression coefficient significantly different from zero at P < 0.05

^a^Breeds: A = Dutch Landrace, B = Dutch Large White, X = F1 cross of A and B

^b^Hetbreed is the model with heterozygosity within breed

^c^Standardised regression coefficients are regression coefficients divided by phenotypic standard deviation

For the synthetic sire line (Table 7), estimates of regression coefficients on heterozygosity were not in a favourable direction (relative to the breeding goal) for all traits. For example, on the one hand, LD100, DP, IMF, PHL, and DRIP (see legend of Table 7 for abbreviations) had regression coefficient estimates that were in the opposite direction to what would be desired for the trait. However, only the estimate for PHL was significantly different from zero. On the other hand, 8 out of 13 traits had regression coefficient estimates that were in a favourable direction. Regression coefficient estimates differed between lines (Model 3), especially for traits with few observations, but were generally in the same direction for both pure lines. Regression coefficient estimates on expected heterosis (Model 4) were small.

**Table 7:** Estimates of regression coefficients (SE) on heterozygosity and expected heterosis for dataset 3 for Models 2, 3, and 4

Trait^a^	Heterozygosity	Hetbreed^b^ Line 1	Hetbreed F1^c^	Hetbreed backcross^d^	Hetbreed Line 2	Expected heterosis
W21, kg	2.80* (0.75)	2.21 (1.63)	− 23.08* (9.13)	− 3.38 (18.20)	2.54* (0.89)	0.01 (0.00)
W150, kg	82.93* (6.02)	82.76* (7.11)	145.03 (88.57)	NA	76.06* (12.82)	0.19 (0.03)
BF100, mm	− 0.86 (0.72)	− 1.73 (0.94)	− 4.59 (9.12)	− 3.61 (50.69)	0.56 (1.21)	0.01 (0.00)
LD100, mm	− 2.43 (1.61)	− 3.47 (2.14)	11.42 (18.43)	147.33 (107.35)	− 0.98 (2.50)	0.05 (0.01)
A40, d	− 41.67* (3.41)	− 49.12* (4.30)	9.28 (39.27)	− 27.99 (85.58)	− 32.14* (4.59)	0.08 (0.01)
DTP, d	− 45.50* (3.41)	− 51.86* (4.62)	− 65.89 (43.97)	78.26 (101.68)	− 36.28* (5.25)	0.05 (0.09)
TFI, kg	− 52.98* (6.38)	− 70.14* (10.10)	− 79.70 (80.17)	− 44.19 (169.92)	− 35.06* (8.85)	0.16 (0.16)
LMP, %	1.62 (1.87)	NA	− 0.42 (23.24)	29.03 (45.47)	1.82 (2.14)	0.24 (0.06)
DP, %	− 0.90 (1.50)	NA	− 8.83 (18.30)	− 36.62 (35.96)	− 0.76 (1.54)	0.07 (0.09)
IMF, g/100 g	− 0.36 (0.42)	NA	− 0.55 (7.43)	NA	− 0.34 (0.53)	− 0.01 (0.01)
PHL	− 21.50* (11.37)	− 40.60 (35.60)	− 147.60 (166.38)	NA	− 19.47 (12.12)	0.79 (0.15)
DRIP, %	1.95 (1.79)	6.80 (5.69)	− 16.56 (26.66)	NA	1.48 (1.95)	0.01 (0.02)
LB1	6.78* (3.27)	9.82* (4.34)	− 198.96 (241.38)	NA	2.68 (5.07)	0.01 (0.01)

* Regression coefficient significantly different from zero at P < 0.05

^a^W21 = 21 days weight in kg, W150 = 150 days weight in kg, BF100 = backfat at 100 kg measured on live animals in mm, LD100 = loin depth at 100 kg measured on live animals in mm, A40 = age at 40 kg, DTP = days from 40 to 120 kg, TFI = total feed intake from 40 to 120 kg in kg (i.e. feed intake per 80 kg live weight gain), LMP = lean meat percentage, DP = dressing percentage (slaughter weight/live weight), IMF = intramuscular fat percentage measured in the laboratory, PHL = pH of loin, DRIP = drip loss is the percentage loss of water from a piece of loin muscle between 96 h post mortem to 120 h post mortem, LB1 = live born first parity

^b^Hetbreed is the model with heterozygosity within breed

^c^Always less than 300 animals with observations for the trait in F1

^d^The backcross is (Line 1 × F1 only). Less than 30 animals with observations for the trait in backcross

Few of the regression coefficients were significantly different from zero (P < 0.05). These were: W21, W150, A40, DTP, TFI, PHL, and LB1 for Model 2; W150, A40, DTP, TFI, and LB1 for Model 3 for Line 1; W21, W150, A40, DTP, and TFI for Model 3 for Line 2; and only W21 for Model 3 for the F1. For the backcross (Model 3) and for Model 4, none of the regression coefficient estimates were significantly different from zero.

For the synthetic line, the traits that had the largest effect of heterozygosity (based on standardised regression coefficients) were W150, A40, and DTP (see Additional file 3: Table S6). The same traits had the largest effect of heterozygosity for Line 1 and Line 2 (Model 3), although the size of the effect was not equal for these two lines. For the F1 and the backcross (Line 1 × F1), there were too few animals to draw conclusions.

### Prediction accuracy

For Norwegian Landrace, predictive ability increased significantly (P < 0.05) from the Base model when including heterozygosity for total number born and litter weight at 3 weeks, but there was little change for the other traits (Table 8).

**Table 8:** Predictive ability (SE) for Norwegian Landrace with and without heterozygosity in the model

Trait	Base model	Model with heterozygosity
Total number born	0.227 (0.014)^a^	0.244 (0.014)^b^
Stillborn	0.161 (0.014)^a^	0.160 (0.014)^a^
Dead at 3 weeks	0.168 (0.014)^a^	0.168 (0.014)^a^
Litter weight 3 weeks, kg	0.342 (0.013)^a^	0.350 (0.013)^b^
Litter variance at 3 weeks	0.143 (0.014)^a^	0.142 (0.014)^a^
Shoulder lesions of sow at weaning	0.273 (0.014)^a^	0.273 (0.014)^a^
Body condition score of sow at weaning	0.243 (0.014)^a^	0.244 (0.014)^a^

^a,b^Different superscript letters within a row indicate significant differences at P < 0.05

For Dutch Landrace, Large White, and their F1 cross, the model that yielded the highest predictive ability varied between traits and breeds (Table 9). Crossbreds benefitted little from including heterozygosity in the model and there were no significant differences (in prediction accuracy) between models. For Dutch Landrace, gestation length benefitted from including heterozygosity, both for Models 2 and 3. For Large White, all three traits had significantly better predictive ability when including heterozygosity compared to the Base model. Predictive ability from including Hetbreed (Model 3) did not differ much from the model with heterozygosity fitted across breeds (Model 2), although significant differences between Models 2 and 3 were found for gestation length in Dutch Landrace. The Hetbreed model had a significantly lower predictive ability than the Base model for total number of born in Dutch Landrace.

**Table 9:** Predictive ability (SE) for Dutch Landrace, Large White, and their F1 cross for the Base model and when including heterozygosity or Hetbreed

Breed	Trait^a^	Base	Heterozygosity	Hetbreed^b^
Dutch Landrace	TNB	0.323 (0.021)^c^	0.319 (0.021)^c,d^	0.318 (0.021)^d^
	LB	0.245 (0.022)^c^	0.247 (0.022)^c^	0.245 (0.022)^c^
	GL	0.499 (0.019)^c^	0.510 (0.019)^d^	0.514 (0.019)^e^
Large White	TNB	0.282 (0.021)^c^	0.293 (0.021)^d^	0.295 (0.021)^d^
	LB	0.254 (0.022)^c^	0.267 (0.022)^d^	0.269 (0.022)^d^
	GL	0.574 (0.018)^c^	0.576 (0.018)^d^	0.575 (0.018)^d^
F1 cross	TNB	0.361 (0.034)^c^	0.360 (0.034)^c^	0.361 (0.034)^c^
	LB	0.282 (0.035)^c^	0.283 (0.035)^c^	0.283 (0.035)^c^
	GL	0.595 (0.029)^c^	0.597 (0.029)^c^	0.598 (0.029)^c^

^a^TNB = total number born, LB = live born, GL = gestation length

^b^Hetbreed is the model with heterozygosity within breed

^c,d,e^Different superscript letters within row indicates significant differences at P < 0.05

For the synthetic line (dataset 3), the predictive ability significantly (P < 0.05) increased for six out of 13 traits when heterozygosity was in the model (Model 2) compared to the Base model (Table 10), although the estimate of the regression coefficient on heterozygosity did not significantly differ from zero for one of these traits, DP. For two traits, TFI and LB1, the model with heterozygosity had significantly lower predictive ability than the Base model. Predictive abilities for Models 3 and 4 were heavily confounded with line origin and are, therefore, presented separately for each line combination (Line 1, Line 2 and F1) (Table 10). The results for the backcross are not presented, since there were too few animals for reliable predictions. For Line 1, Model 3 had a significantly lower predictive ability than the Base model for W150 and A40, while for Line 2, Model 3 had greater predictive ability than the Base model for DTP, TFI, LMP, DP, PHL, and DRIP, but this was significant only for TFI. Compared to the Base model, Model 4 (Line 1) had a significantly higher predictive ability for BF100 but was significantly worse for W150 and TFI. Compared to Model 2, Model 4 had significantly lower predictive ability for W150 and A40 in Line 1, but was not significantly different for any trait for Line 2. However, Model 4 had significantly lower predictive ability than the Base model for W21 and W150 in Line 2. In the F1 cross, Model 4 was not significantly better than the Base model for any trait but had significantly lower predictive ability for LD100, A40, and TFI.

**Table 10:** Predictive ability for the synthetic line for Models 1, 2, 3 and 4

Trait^a^	Model 1	Model 2	Model 3 line 1	Model 3 line 2	Model 3 F1^b^	Model 4 line 1	Model 4 line 2	Model 4 F1^b^
W21, kg	0.140 (0.018)^c^	0.173 (0.018)^d†^	0.065 (0.045)^NS^	0.137 (0.021)^c,e§^	0.070 (0.071)^NS#^	0.044 (0.045)^NS^	0.131 (0.021)^d,e^	0.038 (0.071)^NS^
W150, kg	0.397 (0.016)^c^	0.456 (0.015)^d†§^	0.344 (0.019)^d^	0.336 (0.031)^c,d§^	0.294 (0.079)^c,d#^	0.330 (0.019)^e^	0.330 (0.031)^d^	0.272 (0.080)^c,d^
BF100, mm	0.466 (0.013)^c#^	0.467 (0.013)^c,d†^	0.485 (0.018)^c,d†^	0.444 (0.020)^c,d^	0.422 (0.058)^c,d^	0.484 (0.018)^d^	0.444 (0.020)^c,d^	0.425 (0.058)^c,d#^
LD100, mm	0.611 (0.012)^c^	0.611 (0.012)^c,d^	0.550 (0.018)^c,d^	0.464 (0.019)^c,d^	0.484 (0.052)^c,d#^	0.551 (0.018)^d†^	0.464 (0.019)^c,d^	0.482 (0.052)^d^
A40, d	0.391 (0.014)^c^	0.445 (0.014)^d§^	0.313 (0.020)^e†^	0.377 (0.025)^c,d^	0.232 (0.057)^c,d#^	0.300 (0.020)^c^	0.364 (0.025)^c,d^	0.228 (0.057)^d^
DTP, d	0.324 (0.015)^c^	0.341 (0.015)^d†§^	0.313 (0.020)^c,d†^	0.423 (0.025)^c,d§^	0.276 (0.057)^d#^	0.309 (0.020)^c,d^	0.418 (0.025)^c,d^	0.248 (0.058)^c^
TFI, kg	0.369 (0.017)^c^	0.356 (0.017)^d,e§#^	0.218 (0.026)^c,d†^	0.379 (0.026)^d^	0.253 (0.065)^c,d#^	0.210 (0.026)^d^	0.372 (0.026)^c,e^	0.242 (0.065)^d^
LMP, %	0.614 (0.021)^c#^	0.615 (0.021)^d§^	NA	0.620 (0.023)^c§^	0.524 (0.074)^c,d#^	NA	0.619 (0.023)^c,d^	0.524 (0.074)^c,d#^
DP, %	0.448 (0.024)^c^	0.452 (0.024)^d§^	NA	0.454 (0.026)^c,d^	0.305 (0.082)^c,d#^	NA	0.453 (0.026)^c,d^	0.304 (0.083)^c,d^
IMF, g/100 g	0.595 (0.034)^c^	0.595 (0.034)^c§#^	NA	0.582 (0.035)^c§^	0.653 (0.114)^c#^	NA	0.581 (0.035)^c^	0.652 (0.114)^c^
PHL	0.406 (0.035)^c§^	0.405 (0.035)^c^	0.069 (0.097)^NS†^	0.474 (0.038)^c^	0.142 (0.149)^NS#^	0.059 (0.097)^NS^	0.477 (0.038)^c§^	0.112 (0.150)^NS^
DRIP, %	0.444 (0.034)^c§^	0.443 (0.034)^c^	0.277 (0.094)^c†^	0.473 (0.038)^c^	0.235 (0.146)^NS#^	0.234 (0.095)^c^	0.474 (0.038)^c§^	0.203 (0.148)^NS^
LB1	0.121 (0.042)^c§^	0.085 (0.042)^d^	0.066 (0.079)^NS†^	0.096 (0.049)^c,d^	NA	0.032 (0.079)^NS^	0.103 (0.049)^c,d§^	NA

^NS^Correlation not significantly different from zero

Model 1 = Base model, Model 2 = model with heterozygosity, Model 3 = model with heterozygosity within breed/line, Model 4 = model with expected heterosis level

The highest predictive ability within each line is marked as follows: ^†^ line 1, ^§^ line 2, # = F1. If no correlations are marked within line, all correlations were equal. Significant differences are not taken into account for these indications

^a^W21 = 21 days weight in kg, W150 = 150 days weight in kg, BF100 = backfat at 100 kg measured on live animals in mm, LD100 = loin depth at 100 kg measured on live animals in mm, A40 = age at 40 kg, DTP = days from 40 to 120 kg, TFI = total feed intake from 40 to 120 kg in kg (i.e. feed intake per 80 kg live weight gain), LMP = lean meat percentage, DP = dressing percentage (slaughter weight/live weight), IMF = intramuscular fat percentage measured in the laboratory, PHL = pH of loin, DRIP = drip loss is the percentage loss of water from a piece of loin muscle between 96 h post mortem to 120 h post mortem, LB1 = live born first parity

^b^Always less than 300 animals with observations for the trait in F1

^c,d,e^Differences in superscript letters within row indicate significant differences at P < 0.05. Models 3 and 4 are only comparable to each other within line

### Rank correlations between EBV

Rank correlations differed between models and traits. For Norwegian Landrace, rank correlations of the top 100 selection candidates ranged from 0.79 to 0.83 when comparing Models 1 and 2 (results not shown). The greatest re-ranking was for total number born, litter weight at 3 weeks, and body condition score of the sow at weaning. For Dutch Landrace, Dutch Large White, and their F1 cross (dataset 2), rank correlations between Models 1 and 2 ranged from 0.96 to 0.98, with gestation length having the least re-ranking. Between the Base model and Hetbreed, rank correlations ranged from 0.93 to 0.97, and between Models 2 and 3, rank correlations were 0.98 for all traits. For the synthetic line (dataset 3), there was no re-ranking between the Base model and Model 4 (expected heterosis). Between the Base model and Model 2, rank correlations ranged from 0.72 to 1.00, with TFI and DTP having the lowest rank correlation, and no re-ranking for DP. Between Models 2 and 3, rank correlations ranged from 0.83 to 1.00, with TFI having the lowest rank correlation.

## Discussion

This study investigated the effect of heterozygosity on traits in purebreds, crossbreds, and a synthetic breed. Results show that regression coefficients were large for some traits and for these traits, including heterozygosity in the model led to an increase in the accuracy of genomic prediction of genetic values.

### Regression coefficients on heterozygosity

Estimates of regression coefficients on heterozygosity were large for some traits, such as total number born, litter weight at 3 weeks, weight at 150 days, age at 40 kg, days from 40 to 120 kg, and total feed intake (per 80 kg live weight gain). Theory suggests that heterozygosity has a large effect if the genes show directional dominance (or inbreeding) effects [1]. However, all traits under selection in livestock species can be affected by inbreeding depression [19]. For the traits with a favourable direction of the regression coefficient, heterosis and/or dominance effects are usually reported [2, 20, 21]. These traits are also expected to be negatively affected by inbreeding, since inbreeding increases homozygosity [22, 23]. However, one study found no correlation between heterozygosity and (pedigree) inbreeding coefficients [24], but this study used few markers to estimate heterozygosity and there was little variation in the pedigree-based inbreeding coefficients that were used. In addition, genomic inbreeding coefficients would be more realistic to use, since they are based on actual genotypes and reflect the actual level of heterozygosity in the genome. For traits with unfavourable regression coefficient estimates (e.g. LD100, DP, IMF, PHL, and DRIP), the estimates were generally small relative to the phenotypic standard deviation of the trait [see Additional file 3: Table S6] and of these, only PHL had an estimate that was significantly different from zero. We did not find any reported effects of dominance, heterosis, or inbreeding depression in the literature for the traits with unfavourable regression coefficient estimates on heterozygosity.

Of the traits that were most affected by heterozygosity in the current study, dominance or heterosis effects have been reported for average daily gain, weight at various ages, gestation length, and litter weight [2, 3, 20, 21, 25, 26]. Negative effects of inbreeding have also been reported for daily gain, weight at 90 d, and litter size (total number of born and live born) [22, 27, 28]. However, heterosis and dominance have also been reported for traits for which heterozygosity did not have a large effect in our study, such as backfat [3], although others did not identify heterosis for backfat [21]. This discrepancy in results between studies could be due to an inconsistent sign of the dominance effects across genes, i.e. dominance effects not being directional across loci [1]. The sign of the regression coefficient estimates was generally in agreement with the effect of heterosis in other studies. For example, a positive effect of heterosis on litter weight at 14 days and at weaning [20], and individual weight at all ages [21] have been reported, which is in agreement with the sign of the regression coefficient estimates for LW3, W21 and W150. In addition, positive heterosis effects have been reported for average daily gain from birth to weaning, for birth weight, and for 200-day weight in cattle, and for birth weight, weaning weight, and post-weaning ADG in pigs [26, 29], which agrees with the sign of the regression coefficient estimates for the weight traits and DTP in this study. High heterozygosity reduced feed intake (TFI) in the current study, which is in agreement with some studies [30], although others [31] found no effect of heterosis on feed efficiency. Cassady et al. [20] found that direct heterosis decreased gestation length, which is in agreement with the estimate of the regression coefficient on heterozygosity for gestation length in the current study. However, older studies found no effect of heterosis on gestation length [26].

The trait that was most affected by heterozygosity differed between breeds (dataset 2); for Large White and the crossbreds, number of piglets born alive was most affected by heterozygosity, while for Dutch Landrace gestation length had the largest effect of heterozygosity. For all traits, the estimate of the regression coefficient for CB was closer to the estimate for the parental breed with the largest regression coefficient for PB. This may be due to lack of segregation of the relevant allele(s) for the trait in one breed, such that the CB regression coefficients are closer to those of the breed in which the allele(s) is segregating. However, only the SNPs that segregated in all breeds were used in the analysis. In addition, if heterozygosity does not have the same effect for all breeds, it may not be reasonable to expect the regression coefficients for CB to be the mean of the parental breeds. For the one trait that was the same between datasets 1 and 2, total number of born, estimates of within-breed regression coefficients differed between breeds (Norwegian Landrace, Dutch Landrace, Large White and crossbreds of the latter two). This indicates that heterozygosity does not have the same effect in all breeds, which is in agreement with studies that have found breed differences in dominance [2, 3] and inbreeding effects [4]. This suggests that Model 3 may be more appropriate than Model 2 when analysing multiple breeds.

The above discussion, comparing the results of studies on heterosis [2, 20, 21, 25, 26] and heterozygosity (this study) implies that heterozygosity does not always lead to heterosis. This is logical, because an individual that is heterozygous at loci that do not show directional dominance would not have any advantage over a homozygous individual. Directional dominance is necessary for both heterosis and for non-zero regression coefficients on heterozygosity. Thus, including heterozygosity in the model is expected to improve prediction only for traits for which directional dominance is present. However, there could be dominance effects for a trait without seeing an effect of heterozygosity, because genome-wide heterozygosity may not be a good estimate of dominance effects for traits that are affected by few SNPs or when most animals are homozygous at the relevant loci for the trait.

### Accuracy of genomic prediction

Across the datasets, including heterozygosity in the model generally increased predictive ability for traits that had large regression coefficient estimates, with some exceptions [TFI, LB1 (predictive ability decreased for both despite high regression coefficient), and DP in the synthetic line (predictive ability increased in spite of a small and non-significant regression coefficient)]. These exceptions may be an indication that including heterozygosity in the model may cause noise that results in lower prediction accuracy, although this could also be a result of the complex structure of the datasets used in this study. For dataset 2, there was little difference in predictive ability between the model with heterozygosity across breeds (Model 2) and the model with heterozygosity within breed (Model 3), although the regression coefficient estimates differed between these two models. This suggests that using within-breed instead of across-breed regression coefficients does not consistently improve prediction accuracy. However, each breed had relatively few animals, which makes estimates of regression coefficients less accurate. Since the breeds had quite similar levels of heterozygosity, combining them into a single analysis (Model 2) may result in more accurate regression coefficient estimates, and thus more accurate prediction than within-breed estimates (Model 3).

For CB (dataset 2), including heterozygosity in the model did not significantly improve predictive ability, in contrast to previous studies that found that including inbreeding depression in the model increased predictive ability for total number born in crossbreds [4]. The lack of change in predictive ability between models for CB in the current study may be due to the low variation in heterozygosity within CB animals, which has a direct effect on prediction accuracy. However, variances in heterozygosity were also low in the other breeds analysed and they showed an effect on predictive ability of including heterozygosity in the model. One potential problem with predicting the genetic value of CB, is that there were very few CB animals in the training population when CB animals were in the validation set and, thus, they were mainly predicted based on PB data. In addition, the PB parents of the CB were very homogeneous in heterozygosity, which would make the CB homogeneous in heterozygosity as well, which limits increases in prediction accuracy from including heterozygosity in the model.

For the synthetic line (dataset 3), the interpretation of the comparison between models is a little complicated. For the Base model and for the heterozygosity model (columns 1 and 2 of Table 10), the predictive ability is based on all validation animals from all lines, while the remaining columns are based on animals that belong to each of the lines (or crosses). However, the pairwise comparisons of predictive ability (denoted with superscript letters in Table 10) are based on the subset of animals that belong to each line. This means that not all pairwise comparisons are logical based on the correlations presented. For example, for W150, the predictive ability was highest for Model 2, followed by Model 1 and Model 3 (Line 1). However, in the pairwise comparisons, the predictive ability of Model 2 was not significantly different from that of Model 3 but the predictive ability of both Models 2 and 3 was significantly different from that of Model 1. This can be explained by the fact that the correlations for Models 1 and 2 were based on the entire validation set (across lines), while the correlation for Model 3 (Line 1) was based on validation animals from only one line. Focusing on the subset of Line 1 animals only, Models 2 and 3 showed nearly identical predictive ability (results not shown), while Model 1 had a slightly lower predictive ability. The same phenomenon as described above is observed also for the other pairwise comparisons within line in the table.

### Rank correlations

A low rank correlation indicates more re-ranking of the top animals, which means that the choice of model affects which animals are selected, and indicates that there is an effect of heterozygosity for the trait. The traits with the most re-ranking between Models 1 and 2 across datasets were TNB, LW3, BCS, TFI, and DTP. These traits also had relatively high regression coefficients on heterozygosity. However, prediction accuracy for TFI decreased significantly when including heterozygosity, which suggests that the re-ranking is not necessarily a result of more accurate prediction. That is, re-ranking may occur also if the predictive ability of the “new” model that is being tested is significantly lower than the predictive ability of the existing model (e.g. Base model). For dataset 2, there was little re-ranking between Models 1 and 2. The rank correlations between Models 1 and 3 were somewhat lower than between Models 1 and 2, which indicates that Model 3 had a larger effect than Model 2 on the ranking of the animals when compared to Model 1. Since the predictive ability for Models 2 and 3 were not that different, this may suggest a larger effect of heterozygosity on variation in EBV within breed than between breeds, which leads to re-ranking of animals. However, this was not reflected by significant differences in predictive ability between the models for most traits. In general, re-ranking is only of interest if accuracy improves, as this can inform the decision on whether to implement a new model or not. A small increase in accuracy that does not result in selection of different animals (no re-ranking) may indicate that the gain from the new model is not large enough to justify implementation.

### Potential use of heterozygosity

Implementing the use of heterozygosity in the model when all animals are genotyped should be straightforward with GBLUP, SNP-BLUP and Bayes-type methods [32]. However, most commercial pig breeding companies use single-step (ss-)GBLUP in their breeding programs because not all animals are genotyped. It is more difficult to include heterozygosity in ssGBLUP, because heterozygosity is not available for ungenotyped animals and missing values are not permitted for fixed regressions. Thus, heterozygosity would need to be estimated for ungenotyped animals by segregation analysis [33]. Alternatively, inbreeding coefficients can be estimated from the diagonal of the **H**-matrix, i.e. the combined genomic and pedigree relationship matrix [34]. Since genomic inbreeding is very closely related to heterozygosity, this would be an alternative measure to include in the model.

In addition to including heterozygosity in the model for the estimation of breeding values (as investigated in this study), heterozygosity also has the potential to be used in mate selection in order to maximize heterozygosity in the offspring [35]. This could be achieved by selecting parents that are opposite homozygotes for either as many loci as possible or for the relevant alleles for the trait of interest. This could increase performance of offspring for traits that show a positive effect of heterozygosity, such as litter weight, total number born, weight at 150 days and age at 40 kg. An alternative when producing crossbreds would be to select PB lines with different allele frequencies to maximize heterozygosity in the CB.

### Generality of findings

Our findings should be applicable also to other species for traits that show dominance effects, since inbreeding depression is not specific to species, but rather to a population or a breed [19]. However, it is unlikely that inclusion of heterozygosity is useful for traits that do not show any dominance effects. In livestock production sectors that use crossbreeding, such as for pigs, poultry, and beef cattle, it is necessary to quantify the effect of heterozygosity in order to justify crossbreeding directed at increasing heterozygosity. Even without an effect of heterozygosity, breed complementarity is an important reason for crossbreeding. In addition, it is useful to know whether the effects of heterozygosity are breed-specific in order to direct breed choice in crossbreeding systems. For production sectors that generally do not use crossbreeding, such as dairy cattle and aquaculture, it would be useful to quantify the effect of heterozygosity in order to evaluate how much is gained by setting-up a systematic crossbreeding-based production system.

## Conclusions

Some traits showed large effects of heterozygosity on the phenotype, which is in agreement with other studies that have found heterosis and/or dominance effects for these traits. In addition, for maternal traits, the sign of the regression coefficients for heterozygosity indicated a favourable effect of heterozygosity for these traits, whereas for production, meat and slaughter quality traits, this pattern was less clear, possibly because of the complex data structure. Including heterozygosity in the model for genetic evaluation increased the prediction accuracy for traits that showed the largest effect of heterozygosity and, overall, maternal traits benefitted more than production, meat quality, and slaughter quality traits. Different animals would be selected if heterozygosity was included in the model for these traits. In conclusion, it is beneficial to include heterozygosity in the genomic prediction model for traits that show dominance. It is also possible to use heterozygosity for mate planning in order to benefit from more heterozygous offspring.

## Additional files

**Additional file 1: Table S1.** Base models for Norwegian Landrace. **Table S2.** Base models for Dutch Landrace, Large White, and F1 cross. **Table S3.** Base models for the synthetic line. Description: The base models for all traits and datasets in our study based on models from routine evaluations in Topigs Norsvin.

**Additional file 2: Table S4** Descriptive statistics for dataset 2 separated by breed. **Table S5.** Descriptive statistics for dataset 3 separated by line.

**Additional file 3: Table S6.** Regression coefficients divided by phenotypic standard deviation for the synthetic line for Models 2 to 4. Description: Regression coefficient estimates for dataset 3 divided by the phenotypic standard deviation for the trait.
