# Supplementary material for: Effects of heterozygosity on performance of purebred and crossbred pigs
Source: Genet Sel Evol. 2019 Feb 28;51:8. doi: 10.1186/s12711-019-0450-1 (PMC6396501; doi:10.1186/s12711-019-0450-1)
Supplement: Supplementary file 1 — Additional file 1: Table S1. Base models for Norwegian Landrace. Table S2. Base models for Dutch Landrace, Large White, and F1 cross. Table S3. Base models for the synthetic line. Description: The base models for all traits and datasets in our study based on models from routine evaluations in Topigs Norsvin. [file 12711_2019_450_MOESM1_ESM.docx]

**Additional file 1**

**Models**

**Table S1 Base models for Norwegian Landrace**

| **Trait^a^** | **Variables^b,c^** | | | | | | | | | | | | | |
| --- | --- | --- | --- | --- | --- | --- | --- | --- | --- | --- | --- | --- | --- | --- |
|  | **Parity dam** | **Parity** | **Herd-year** | **Season** | **Breed year** | **Age dam *parity** | **Age dam^2^*parity** | **Weighed piglets** | **Age sow** | **Age sow^2^** | **Weaned** | **Litter** | **Per.env.** | **Animal** |
| TNB | F | F | F | F | F | E | E |  |  |  |  | R | R | G |
| SB | F | F | F | F | F | E | E |  |  |  |  | R | R | G |
| D3 | F | F | F | F | F | E | E |  |  |  |  | R | R | G |
| LW3 | F | F | F | F | F | E | E | F |  |  |  | R | R | G |
| VAR3 | F | F | F | F | F | E | E | F |  |  |  | R | R | G |
| SL | F | F | F | F | F | E | E |  | E | E | F | R | R | G |
| BCS | F | F | F | F | F | E | E |  | E | E | F | R | R | G |

^a^TNB = total number born, SB = stillborn, D3 = number of dead piglets at 3 weeks, LW3 = litter weight at 3 weeks in kg, VAR3 = variance in litter weight at 3 weeks, SL = shoulder lesions of the sow at weaning, BCS = body condition score of the sow at weaning.

^b^Parity dam = parity of the dam of the sow, parity = parity of the sow, herd-year = combination of herd and year, season = code for season (1-4), breed year = combination of breed of the sire of the litter and year, age dam*parity = a factor to correct for the age of the dam of the sow within parity, age dam^2^*parity = a factor to correct for the age of the dam of the sow squared within parity, weighed piglets = number of piglets weighed, age sow = age of the sow, age sow^2^ = age of the sow squared , weaned = number of piglets weaned, litter = ID of the litter, per.env. = permanent environment, animal = genetic effect of animal.

^c^F indicates fixed effects, E fixed regressions, R random effects, and G genetic effects.

**Table S2 Base models for Dutch Landrace, Large White, and F1 cross**

| **Variables^a,b^** | **Total number born** | **Live born** | **Gestation length** |
| --- | --- | --- | --- |
| Parity | F | F |  |
| Breed | F | F | F |
| Farm-litter | F | F | F |
| Farrowing quarter | F | F | F |
| Herd-year-season | R | R | R |
| Permanent environment | R | R | R |
| Animal | G | G | G |

^a^Parity = parity of the sow, breed = breed, farm-litter = combination of farm and litter (i.e. pen), farrowing quarter = which quarter (of the year) the sow farrowed in, permanent environment = permanent environment, animal = genetic effect of animal.
^b^F indicates fixed effects, R random effects, and G genetic effects.

**Table S3 Base models for the synthetic line**

| **Trait^a^** | **Variables^b,c^** | | | | | | | | | | | | | | | | | | | | | | | | | |
| --- | --- | --- | --- | --- | --- | --- | --- | --- | --- | --- | --- | --- | --- | --- | --- | --- | --- | --- | --- | --- | --- | --- | --- | --- | --- | --- |
|  | **lgpcode** | **Farm*year** | **Sex** | **HYS** | **Parity dam** | **Birth month** | **Technician** | **Herd compartment** | **Season of birth** | **CT version** | **Liveborn** | **Liverborn^2^** | **Test method** | **Live weight** | **Live weight *CT version** | **Liverweight^2^ * CT version** | **Hot carcass weight** | **Meat size** | **Meat size^2^** | **Avg. Dif. Days driploss** | **Litter** | **Pen** | **Slaughter date** | **NIRS date** | **Animal** | **Dam** |
| W21 | F | F | F |  | F | F |  |  |  |  | E | E |  |  |  |  |  |  |  |  | R |  |  |  | G | G |
| W150 | F |  | F | F | F | F | F |  |  |  | E | E |  |  |  |  |  |  |  |  | R | R |  |  | G |  |
| BF100 | F |  | F | F | F | F |  |  |  |  | E | E | F |  |  |  |  |  |  |  | R | R |  |  | G |  |
| LD100 | F |  | F | F | F | F |  |  |  |  | E | E | F |  |  |  |  |  |  |  | R | R |  |  | G |  |
| A40 | F | F |  |  | F | F |  |  |  |  | E |  |  |  |  |  |  |  |  |  | R |  |  |  | G |  |
| DTP | F | F |  |  | F | F |  | F |  |  | E |  |  |  |  |  |  |  |  |  | R | R |  |  | G |  |
| TFI | F | F |  |  | F | F |  | F |  |  | E |  |  |  |  |  |  |  |  |  | R | R |  |  | G |  |
| LMP | F | F |  |  | F | F |  | F |  |  | E |  |  | E |  |  |  |  |  |  | R | R |  |  | G |  |
| DP | F | F |  |  | F | F |  | F |  | F | E |  |  |  | E | E |  |  |  |  | R | R |  |  | G |  |
| IMF | F | F |  |  | F | F |  | F |  |  | E |  |  |  |  |  | E |  |  |  |  |  |  | R | G |  |
| PHL | F | F |  |  | F | F |  | F |  |  | E |  |  |  |  |  | E |  |  |  |  |  | R |  | G |  |
| DRIP | F | F |  |  | F | F |  | F |  |  | E |  |  |  |  |  | E | E | E | E |  |  | R |  | G |  |
| LB1 | F | F |  |  | F | F |  |  | F |  |  |  |  |  |  |  |  |  |  |  | R |  |  |  | G |  |

^a^W21 = weight at 21d in kg, W150 = weight at 150d in kg, BF100 = backfat depth at 100kg in mm, LD100 = loin depth at 100kg in mm, A40 = age at 40kg in days, DTP = days from 40 to 120kg, TFI = total feed intake from 40-120kg in kg, LMP = lean meat percentage, DP = dressing percentage (slaughter weight/live weight), IMF = intra-muscular fat, PHL = pH of loin, DRIP = percentage loss of water from a piece of loin muscle between 96h post mortem to 120h post mortem, LB1 = live born first litter.

^b^lgpcode = code for original line or line combination, Farm*year = herd-year of birth, Sex = biological sex, HYS = herd-year-season effects, Parity dam = the parity number of the dam of the animal, Birth month = month the animal was born, Technician = code for technician who took the measurement, Herd compartment = housing room within herd, season of birth = season the animal was born, CT version = version of CT scanner used, Liveborn = number of piglets born alive, Liveborn^2^ = number of piglets born alive squared, Test method = trait measurement method, live weight = live weight of animal, Live weight*CT version = live weight combined with CT version, Live weight^2^*CT version = live weight squared combined with CT version, Hot carcass weight = weight of carcass while warm, Meat size = size of the meat sample in grams, Avg. Dif. Days driploss = average interval from slaughter to meat quality testing in days, Litter = ID of the litter, NIRS date = date of Near InfraRed Spectroscopy test, Animal = genetic effect of animal, Dam = genetic effect of dam.

^c^F indicates fixed effects, E fixed regressions, R random effects, and G genetic effects.
