# Supplementary material for: Effects of heterozygosity on performance of purebred and crossbred pigs
Source: Genet Sel Evol. 2019 Feb 28;51:8. doi: 10.1186/s12711-019-0450-1 (PMC6396501; doi:10.1186/s12711-019-0450-1)
Supplement: Supplementary file 2 — Additional file 2: Table S4 Descriptive statistics for dataset 2 separated by breed. Table S5. Descriptive statistics for dataset 3 separated by line. [file 12711_2019_450_MOESM2_ESM.docx]

**Additional file 2**

**Descriptive statistics**

**Table S4 Descriptive statistics for dataset 2 separated by breed^a^**

| **Trait^b^** | **Mean (SD) Breed A** | **Mean (SD) Breed B** | **Mean (SD) Breed X** | **h^2^** |
| --- | --- | --- | --- | --- |
| TNB | 15.21 (3.40) | 16.12 (3.61) | 15.47 (3.60) | 0.10 |
| LB | 13.99 (3.24) | 14.50 (3.35) | 14.52 (3.34) | 0.07 |
| GL, d | 116.13 (1.62) | 115.16 (1.57) | 115.37 (1.56) | 0.34 |

^a^Breed A = Dutch Landrace, Breed B = Large White, Breed X = the F1 cross of breed A and B,

^b^Traits: TNB = total number born, LB = live born, GL = gestation length in days.

**Table S5 Descriptive statistics for dataset 3 separated by line^a^**

| **Trait^b^** | **Mean (SD) Line 1** | **Mean (SD) Line 2** | **Mean (SD) F1** | **Mean (SD) backcross^c^** | **h^2^** |
| --- | --- | --- | --- | --- | --- |
| W21, kg | 6.54 (1.37) | 6.98 (1.42) | 7.02 (1.45) | 6.52 (1.11) | 0.06 |
| W150, kg | 109.13 (12.12) | 108.30 (10.08) | 112.17 (11.35) | NA | 0.36 |
| BF100, mm | 7.60 (1.52) | 6.39 (2.77) | 5.75 (1.69) | 3.98 (0.76) | 0.43 |
| LD100, mm | 58.71 (4.90) | 41.65 (3.36) | 50.63 (7.21) | 45.90 (1.72) | 0.37 |
| A40, d | 86.13 (7.36) | 87.63 (6.65) | 82.74 (7.39) | 80.16 (4.19) | 0.41 |
| DTP, d | 75.99 (10.80) | 79.20 (7.95) | 72.48 (8.09) | 73.36 (7.18) | 0.42 |
| TFI, kg | 170.29 (19.78) | 174.59 (13.61) | 173.28 (13.18) | 176.09 (12.57) | 0.38 |
| LMP, % | NA | 61.41 (3.47) | 61.70 (2.97) | 59.66 (2.65) | 0.59 |
| DP, % | NA | 71.25 (2.58) | 70.53 2.32) | 69.64 (2.11) | 0.27 |
| IMF, g/100 g | NA | 1.78 (0.41) | 1.75 (0.40) | NA | 0.68 |
| PHL | 579.29 (17.69) | 560.28 (12.13) | 552.02 (11.63) | NA | 0.37 |
| DRIP, % | 2.72 (1.21) | 3.90 (1.80) | 4.56 (1.68) | NA | 0.32 |
| LB1 | 8.65 (2.81) | 7.99 (2.89) | 6.80 (3.03) | NA | 0.11 |

^a^Dataset 3 consists of a synthetic sire line made of two lines of the same breed (Line 1 and 2) that have been under selection independently from each other. The dataset contains both animals of the two original lines, their F1 and a backcross (Line 1 x F1).

^b^W21 = 21d weight in kg, W150 = 150d weight in kg, BF100 = backfat at 100kg measured on live animals in mm, LD100 = loin depth at 100kg measured on live animals in mm, A40 = age at 40kg, DTP = days from 40-120kg, TFI = total feed intake from 40-120kg in kg (i.e. feed intake per 80 kg live weight gain), LMP = lean meat percentage, DP = dressing percentage (slaughter weight/live weight), IMF = intramuscular fat percentage measured in the laboratory, PHL = pH of loin, DRIP = drip loss is the percentage loss of water from a piece of loin muscle between 96h post mortem to 120h post mortem, LB1 = live born first parity.

^c^Less than 30 animals with observations for the trait in backcross.
