# Supplementary material for: Effects of heterozygosity on performance of purebred and crossbred pigs
Source: Genet Sel Evol. 2019 Feb 28;51:8. doi: 10.1186/s12711-019-0450-1 (PMC6396501; doi:10.1186/s12711-019-0450-1)
Supplement: Supplementary file 3 — Additional file 3: Table S6. Regression coefficients divided by phenotypic standard deviation for the synthetic line for Models 2 to 4. Description: Regression coefficient estimates for dataset 3 divided by the phenotypic standard deviation for the trait. [file 12711_2019_450_MOESM3_ESM.docx]

**Additional file 3**

**Regression coefficients divided by phenotypic standard deviation**

**Table S6 Regression coefficients divided by phenotypic standard deviation for the synthetic line for Models 2 to 4**

| **Trait^a^** | **Heterozygosity** | **Hetbreed^b^ Line 1** | **Hetbreed F1^c^** | **Hetbreed backcross^d^** | **Hetbreed Line 2** | **Expected heterosis** |
| --- | --- | --- | --- | --- | --- | --- |
| W21, kg | 1.96 | 1.54 | -16.14 | -2.36 | 1.78 | 0.01 |
| W150, kg | 7.09 | 7.07 | 12.40 | NA | 6.50 | 0.02 |
| BF100, mm | -0.39 | -0.79 | -2.09 | -1.65 | 0.26 | 0.01 |
| LD100, mm | -0.26 | -0.37 | 1.21 | 15.57 | -0.10 | 0.00 |
| A40, d | -5.87 | -6.92 | 1.31 | -3.94 | -4.53 | 0.01 |
| DTP, d | -4.69 | -5.35 | -6.80 | 8.07 | -3.74 | 0.00 |
| TFI, kg | -3.26 | -4.32 | -4.90 | -2.72 | -2.16 | 0.01 |
| LMP, % | 0.47 | NA | -0.12 | 8.39 | 0.53 | 0.07 |
| DP, % | -0.35 | NA | -3.44 | -14.25 | -0.29 | 0.03 |
| IMF, g/100g | -0.88 | NA | -1.34 | NA | -0.83 | -0.02 |
| PHL | -1.58 | -2.98 | -10.84 | NA | -1.43 | 0.06 |
| DRIP, % | 1.09 | 3.80 | -9.25 | NA | 0.83 | 0.01 |
| LB1 | 2.37 | 3.43 | -69.57 | NA | 1.00 | 0.00 |

^a^W21 = 21d weight in kg, W150 = 150d weight in kg, BF100 = backfat at 100kg measured on live animals in mm, LD100 = loin depth at 100kg measured on live animals in mm, A40 = age at 40kg, DTP = days from 40-120kg, TFI = total feed intake from 40-120kg in kg (i.e. feed intake per 80 kg live weight gain), LMP = lean meat percentage, DP = dressing percentage (slaughter weight/live weight), IMF = intramuscular fat percentage measured in the laboratory, PHL = pH of loin, DRIP = drip loss is the percentage loss of water from a piece of loin muscle between 96h post mortem to 120h post mortem, LB1 = live born first parity.

^b^Hetbreed is the model with heterozygosity within breed.

^c^Always less than 300 animals with observations for the trait in F1.

^d^The backcross is (Line 1 x F1) only. Less than 30 animals with observations for the trait in backcross.
